# Protective properties of extracellular vesicles in sepsis models: a systematic review and meta-analysis of preclinical studies

**DOI:** 10.1186/s12967-023-04121-7

**Published:** 2023-04-17

**Authors:** Shujun Yang, Kanglong Zhang, Jingyu Hou, Xin Liu, Daishi Xu, Xuxiang Chen, Shuangmei Li, Yinghui Hong, Changqing Zhou, Hao Wu, Guanghui Zheng, Chaotao Zeng, Haidong Wu, Jiaying Fu, Tong Wang

**Affiliations:** 1 Department of Emergency, The Eighth Affiliated Hospital of Sun Yat-sen University, Shenzhen, 518003 Guangdong People’s Republic of China; 2 Department of Emergency, Sun Yat-sen Memorial Hospital of Sun Yat-sen University, Guangzhou, 510120 Guangdong People’s Republic of China

**Keywords:** Extracellular vesicles, Sepsis, Meta-analysis, Preclinical analysis, Mesenchymal stem cells

## Abstract

**Background:**

Multiple preclinical studies have reported a beneficial effect of extracellular vesicles (EVs), especially mesenchymal stem cells derived EVs (MSC-EVs), in the treatment of sepsis. However, the therapeutic effect of EVs is still not universally recognized. Therefore, we conducted this meta-analysis by summarizing data from all published studies that met certain criteria to systematically review the association between EVs treatment and mortality in animal models of sepsis.

**Methods:**

Systematic retrieval of all studies in PubMed, Cochrane and Web of Science that reported the effects of EVs on sepsis models up to September 2022. The primary outcome was animal mortality. After screening the eligible articles according to inclusion and exclusion criteria, the inverse variance method of fixed effect model was used to calculate the joint odds ratio (OR) and 95% confidence interval (CI). Meta-analysis was performed by RevMan version 5.4.

**Results:**

In total, 17 studies met the inclusion criteria. Meta-analysis of those studies showed that EVs treatment was associated with reduced mortality in animal models of sepsis (OR 0.17 95% CI: 0.11,0.26, P < 0.001). Further subgroup analysis showed that the mode of sepsis induction, the source, dose, time and method of injection, and the species and gender of mice had no significant effect on the therapeutic effect of EVs.

**Conclusion:**

This meta-analysis showed that MSC-EVs treatment may be associated with lower mortality in animal models of sepsis. Subsequent preclinical studies will need to address the standardization of dose, source, and timing of EVs to provide comparable data. In addition, the effectiveness of EVs in treating sepsis must be studied in large animal studies to provide important clues for human clinical trials.

**Supplementary Information:**

The online version contains supplementary material available at 10.1186/s12967-023-04121-7.

## Background

Sepsis, caused by an abnormal host response to infection, is a life-threatening organ dysfunction [1]. According to an epidemiological analysis by Kristina E et al., there were 48.9 million cases of sepsis worldwide in 2017, of which 11 million resulted in patient deaths, accounting for about 20% of total global deaths [2]. The treatment of sepsis mainly involves eliminating the source of infection, suppressing the inflammatory response, and following with corresponding symptomatic and supportive treatment [3]. A potential treatment that can help reduce the massive inflammatory process, tissue damage is still lacking. Therefore, it is urgent to find new therapeutic methods to improve the clinical outcome of patients with sepsis.

Mesenchymal stem cells (MSCs) have attracted interest in the past few years based on advantages such as differentiation potential, self-renewal and self-secretion [4]. MSCs has been clinically studied as a therapeutic agent in a variety of diseases such as diabetes, Alzheimer's disease and Osteoarthritis[5–7]. However, there are some challenges with the use of MSC, including low survival rate and difficult to reach injury [8]. In recent years, many preclinical studies have introduced MSC-derived extracellular vesicles into the treatment of various diseases [9–11]. Extracellular vesicles (EVs) can be divided into exosomes, microvesicles, and apoptotic bodies according to their diameters and can come from almost all cells, especially stem cells [12]. EVs can carry and protect specific subsets of proteins, lipids, and genetic material such as mRNA, miRNA, and DNA from the extracellular environment [4, 13]. Compared with cell therapy, EVs have the advantages of low immunogenicity, low toxicity and relative stability in the blood [14].

To date, several studies have focused on EVs for the treatment of sepsis in animals [15–17]. Most of these studies demonstrated that EVs treatment reduced mortality in animal models of sepsis [18–33]; however, individual studies have come to different conclusions [34]. In addition, there is no uniform conclusion regarding EVs with different cell sources, and varying injection doses, routes of administration, and duration of treatment have been used. Therefore, this meta-analysis was conducted to explore the efficacy of EVs in treating sepsis in animal models and to provide the latest evidence support for clinical studies.

## Methods

The Preferred Reporting Items for Systematic Reviews and Meta-Analyses (PRISMA) criteria was used in this meta-analysis [35]. Ethics approval is not required to analyze published articles. The article provides all supporting data, and additional information is supplemented online.

### Data sources and search strategies

A systematic literature review was conducted completely using three databases, including PubMed, Cochrane, and Web of Science, to screen for any in vivo studies investigating the use of EVs for sepsis. Detailed search strategies are shown in Additional file 1. The database was last supplemented on September 23, 2022. Only publications whose language is English are included.

### Inclusion and exclusion criteria

The inclusion criteria were as follows: (1) to evaluate the therapeutic effect of EVs on sepsis animal model; (2) the protective effect of EVs or EVs-derived molecules was the main focus of research; (3) the study involved an animal model of sepsis or endotoxemia; (4) the study reported mortality rates; and (5) The language of the research is English.

The exclusion criteria were as follows: (1) extracellular vesicles were not directly used as therapy; (2) extracellular vesicles were genetically modified; (3) There were other complications in the animal model; (4) no sepsis occurred; (5) lack of end points of interest in the study data; (6) in vitro study; (7) the study was duplicated; (8) studies published in a non-English language; and (9) no original research was performed (e.g., book chapters, reviews, editorials, meta-analysis, etc.)

### Data extraction

Two independent reviewers, YS and ZK, conducted data extraction, and the differences encountered were resolved through discussion between them. The following data were collected: first author, country or region, year of publication, animal type, sex, and number, sepsis model type, origin of EVs cells, dose, injection method, injection time, observation time after EVs administration, and indicators related to the primary outcome. For studies that did not provide the required results, Engauge Digitizer version 10.8 software was used to extract data from accompanying graphs [36].

### Risk of bias

Risk of bias was assessed according to the Systematic Review Center for Laboratory Animal Experimentation (SYRLE) [37]. SYRCLE includes: selection bias, performance bias, detection bias, attrition bias, reporting bias and others. The risk of bias was assessed carefully by two independent reviewers as low risk, high risk, or unclear risk based on the content of the article. Any disputes encountered during the evaluation process were resolved through discussion.

### Statistical analysis

The effect size of this meta-analysis was mortality. The OR values of the EVs treatment group and control group were calculated to determine the combined effect size. The I^2^ statistic was used to analyze heterogeneity. I^2^ > 50% indicated significant heterogeneity [38]. The effect model of meta-analysis was selected according to whether the heterogeneity was significant [39]. All statistical analyses in this manuscript were performed by using fixed-effects model according to the heterogeneity test results. Visual inspection of the funnel plot was used to test whether publication bias existed [40]. Sensitivity analyses were performed by removing each study individually from the results of the meta-analysis. All statistical analyses were performed using RevMan version 5.4. A difference of P < 0.05 (two-sided) was considered statistically significant.

## Results

### Study inclusion

**Fig. 1:**
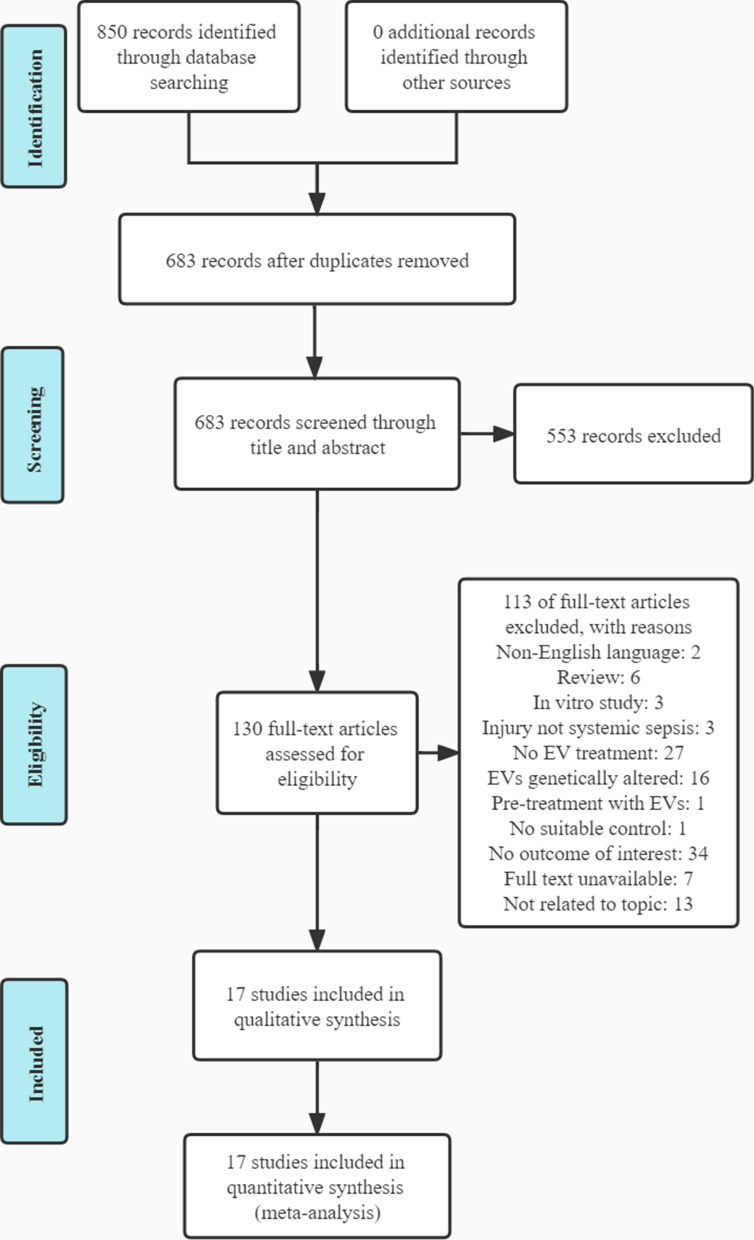
Flow diagram of the study selection

A total of 850 articles were retrieved from the three databases according to the search strategy. After the elimination of 167 duplicate articles, 553 of 683 articles were initially screened and excluded according to the title and abstract. The full text of the remaining 130 articles was reviewed, and 17 articles were selected to be included in this meta-analysis according to the inclusion and exclusion criteria [18–34]. The specific filtering steps are shown in Fig. 1.

### Study characteristics

This meta-analysis ultimately included 17 articles, the basic characteristics of which are shown in Table 1. A total of 395 rodents (rats and mice) were included. Sepsis models were induced mainly by cecal ligation and puncture (CLP) or by intraperitoneal injection of lipopolysaccharide (LPS). EVs were mainly derived from human or mouse bone marrow, adipose mesenchymal tissue, or human umbilical cord blood mesenchymal tissue. Cecal ligation and puncture (CLP) or intraperitoneal injection of lipopolysaccharide (LPS) were used in the sepsis model. The EVs used to treat sepsis are derived mainly from stem cells derived from human cord blood mesenchymal tissue or stem cells from different parts of the mouse body, such as bone marrow or adipose mesenchymal tissue. EVs were mainly injected intravenously or intratracheally into mice within 6 h after induction. The injection dose ranged from 0.05 to1000μg. The follow-up time was between 2 to 7 days.

**Table 1:** General characteristics of preclinical studies investigating the efficacy of extracellular vesicles in sepsis models

Author	Species	Gender	No. of controls	No. of treated animals	Sepsis model	EVs source,Compatibility	Dose	Route of administration	Time of delivery post-sepsis induction	Fluid treatment after modeling	Time to observe results after EVs administration	Control
Chia-Lo et al. (2018)	Rat SD	Male	19	18	CLP	ADSCs, Allogeneic	100 µg	IV	3 h	NR	5 day	NR
Deng et al. (2020)	Mouse C57BL/6	Male	12	12	LPS	BMSCs, Allogeneic	100 μg	IT	1 h	NR	7 day	PBS
Miksa et al. (2006)	Rat SD	Male	16	16	CLP	DCs, Allogeneic	1,000 μg	IV	0 or 5 h	NS	10 day	PBS
Huimin et al. (2022)	Mouse C57BL/6	Male	12	12	LPS	ADMSCs, Xenogenic	100 μg	IT	1 h	NR	7 day	PBS
Xiaoyan et al. (2022)	Mouse C57BL/6	Male	15	15	CLP	ADMSCs, Allogeneic	2 μg/kg	IV	4 h	Imipenem	7 days	PBS
Yueet al. (2018)	Mouse CD-1 outbred	Either sex	15	23	CLP	EPCs, Xenogenic	2 mg/kg	IV	4 h	Imipenem	7 days	PBS
Dongxuan et al. (2022)	Mouse C57BL/6	Male	7	6	CLP	AECs, Xenogenic	1 × 10^8^	IV	0 h	NR	7 days	PBS
Danyang et al. (2021)	Rat SD	Either sex	16	16	CLP	MSC, Allogeneic	2 × 10^7^	IV	UR	PBS	2 days	PBS
Jia et al. (2021)	Mouse C57BL/6	Male	10	10	CLP	BMMSCs, Allogeneic	1.5 × 10^9^	NR	4 h	NR	7 days	PBS
Qin et al. (2021)	Mouse C57BL/6	Either sex	10	10	CLP	MSCs, Xenogenic	2 μg/g	NR	0 h or 6 h	NR	10 days	NR
Yuhan et al. (2022)	Mouse C57BL/6 J	Either sex	6	6	CLP	RBC, Xenogenic	10 μg	IV	2 h	NR	2 days	PBS
Xiaohong et al. (2015)	Mouse C57BL/6	Male	8	8	CLP	BMMSCs, Allogeneic	2 μg/g	IV	1 h	NS	3 days	PBS
Jie et al. (2021)	.Mouse C57BL/6	Male	10	10	CLP	MSCs, Xenogenic	30 UL	IV	6 h	NS	7 days	PBS
Rongxue et al. (2020)	Mouse C57BL/6	Male	12	12	CLP	MSC, Xenogenic	120 ug	IV	3 h	NS	3 days	PBS
Yuan et al. (2021)	Mouse C57	Male	15	15	LPS	BMSC, Allogeneic	100 μg	IV	1 h	NR	2 days	PBS
Fang et al. (2020)	Mouse C57/BL6	Either sex	6	6	CLP	AMSCs, Xenogenic	100 μg	IV	4 h	NR	2 days	PBS
Mahshid et al. (2021)	Mouse C57/BL6	Either sex	8	8	E. coli	ucMSCs, Xenogenic	1.5 μg/g	IV	5 h	NR	7 days	PBS

*CLP* Cecal ligation and puncture, *IV* Intravenous, *IT* Intratracheal injection, *LPS* Lipopolysaccharide, *NR* Not reported, *NS* Normal saline, *PBS* Phosphate buffered saline, *SD* Sprague Dawley, *E. coli* Escherichia coli, *ADSCs* Adipose-derived mesenchymal stem cells, *BMMSCs* Bone marrow mesenchymal stem cells. *EPCs* Endothelial progenitor cells, *RBC* Red blood cell, *DCs* dendritic cells, *ADMSCs* Adipose-derived mesenchymal stem cells; *AECS* Amnion epithelial cells, *AMSC* Amniotic membrane stem cell, *ucMSCs* Umbilical cord-derived mesenchymal stem cells

### Risk of bias assessment

The risk of bias among included studies was assessed by SYRCLE's RoB tool (Table 2). All of the studies were considered to have RoB risks. Although 10 studies (58%) reported randomization of animals, none showed how random sequences were generated or whether assignments were adequately concealed. Therefore, the RoB scores in the selection bias component were "unclear" for all studies. None of the studies mentioned whether the animals were raised and evaluated randomly, or whether the researchers were blind to the animal intervention program. Five studies [18, 24–26, 32] mentioned that assessors were blinded to the animal interventions. Three studies [24, 28, 30] did not have sufficient outcome data. All studies had a low risk of reporting bias. One study [23] may have had a problem with the experimental design, which resulted in a RoB score of "high risk". Otherwise, no significant issues concerning bias were identified that were not covered in the SYRCLE’s RoB tool.

**Table 2:** SYRCLE Risk of Bias Assessment for included studies

Author	Random sequence generation?	Groups similar at baseline?	Allocation concealed?	Animals randomly housed?	Blinding of caregivers and/or examiners?	Random selection for outcome assessment?	Blinding of outcome assessor?	Incomplete outcome data addressed?	Free from selective outcome reporting?	Free from other bias?
Chia-Lo et al. (2018)	U	U	U	U	U	U	L	U	L	L
Deng et al. (2020)	U	U	U	U	U	U	U	U	L	L
Miksa et al. (2006)	U	U	U	U	U	U	U	U	L	L
Huimin et al. (2022)	U	U	U	U	U	U	U	U	L	L
Xiaoyan et al. (2022)	U	U	U	U	U	U	U	U	L	H
Yueet al. (2018)	U	U	U	U	U	U	L	H	L	L
Dongxuan et al. (2022)	U	U	U	U	U	U	L	U	L	L
Danyang et al. (2021)	U	U	U	U	U	U	U	H	L	L
Jia et al. (2021)	U	U	U	U	U	U	U	H	L	L
Qin et al. (2021)	U	U	U	U	U	U	U	U	L	L
Yuhan et al. (2022)	U	U	U	U	U	U	U	U	L	L
Xiaohong et al. (2015)	U	U	U	U	U	U	U	U	L	L
Jie et al. (2021)	U	U	U	U	U	U	L	U	L	L
Rongxue et al. (2020)	U	U	U	U	U	U	U	U	L	L
Yuan et al. (2021)	U	U	U	U	U	U	U	U	L	L
Fang et al. (2020)	U	U	U	U	U	U	L	U	L	L
Mahshid et al. (2021)	U	U	U	U	U	U	U	U	L	L

*H* High risk of bias, *L* Low risk of bias, *U* Unclear risk of bias

### Effects of extracellular vesicles on sepsis

**Fig. 2:**
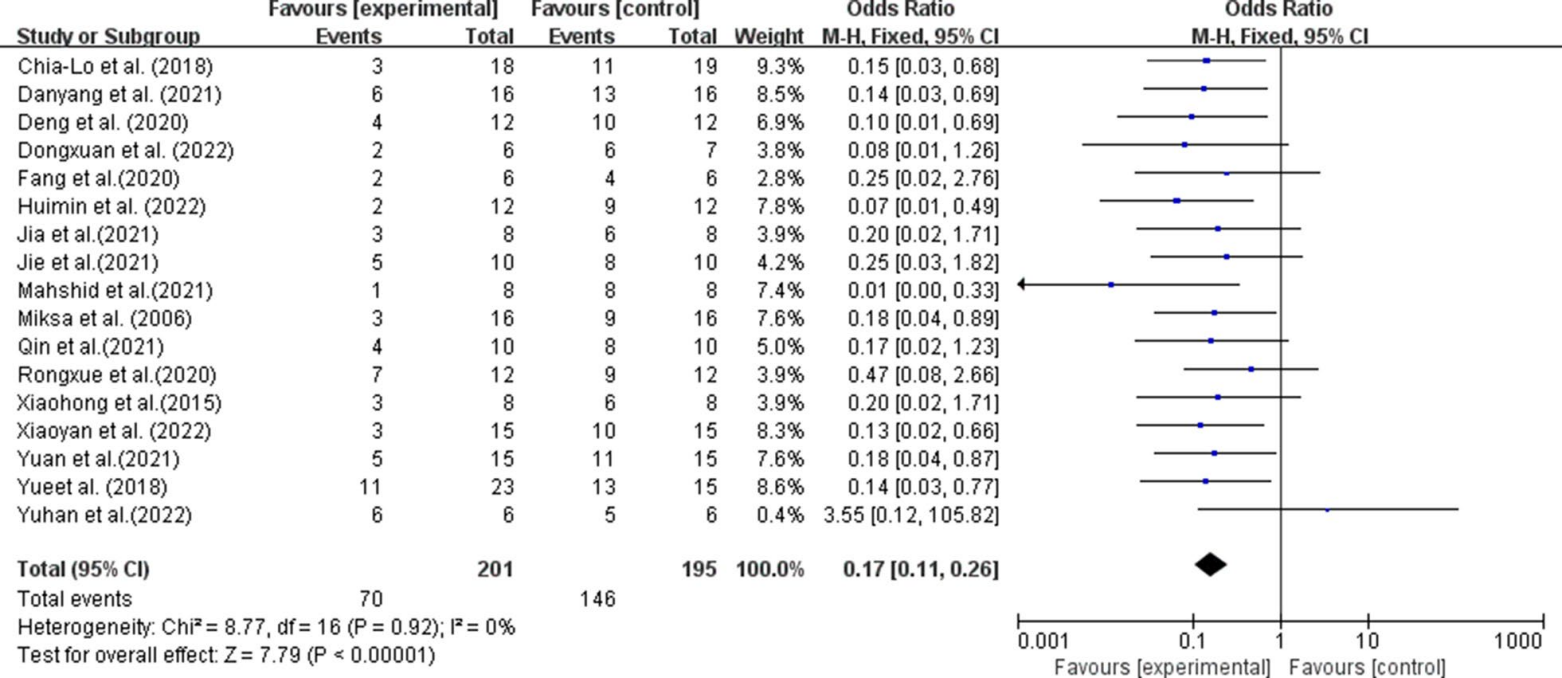
Forest plots summarize the effect of extracellular vesicles therapy on mortality in a preclinical model of sepsis

A total of 17 reports related to EVs treatment of sepsis were included in this study, all of which reported mortality. Mortality at the end point was 146 of 195 (74.9%) in the control group and 70 of 201 (34.8%) in the EVs-treated group. As shown in Fig. 2, results analysis showed that EVs treatment significantly reduced sepsis mortality (OR 0.17, 95% CI: 0.11, 0.26, P < 0.001). Sensitivity analysis was conducted by excluding each study from the results of meta-analysis. The results showed that reducing any one study did not make a significant difference to the results.

### Subgroup analysis

Subgroup analyses were performed to evaluate the efficacy of EVs in the treatment of sepsis, taking into account the generality and reproducibility of treatment outcomes across different experimental conditions. The use of EVs in rats (OR = 0.37, 95% CI: 0.22–0.62, P < 0.001) was more effective than in mice (OR = 0.49, 95% CI: 0.39–0.61, P < 0.001) (Additional file 1: Fig. S1). Animals that did not limit their sex (OR = 0.19, 95% CI: 0.11–0.33, P < 0.001) had higher survival rates than those that used only male animals (OR = 0.14, 95% CI: 0.06–0.29, P < 0.001) (Additional file 1: Fig. S2). Compared with the CLP model (OR = 0.51, 95% CI: 0.41–0.64, P < 0.001), EVs had better therapeutic outcomes in non-CLP models (OR = 0.32, 95% CI: 0.20–0.53, P < 0.001) than in CLP models (Additional file 1: Fig. S3).

Among the 17 studies, EVs in 13 of them were derived from MSCs (OR = 0.15, 95% CI: 0.09–0.26, P < 0.001), which had a better therapeutic effect on sepsis compared with EVs derived from other cells (OR = 0.21, 95% CI: 0.08–0.55, P < 0.001) (Additional file 1: Fig. S4). The most common method of administration of EVs was intravenous injection (N = 12; 70.6%). Subgroup analysis showed that intratracheal administration (OR = 0.08, 95% CI: 0.02–0.33, P < 0.001), in comparison with intravenous injection (OR = 0.19, 95% CI: 0.11–0.32, P < 0.001), was more likely to improve survival (Additional file 1: Fig. S5). Doses and units of injected EVs varied widely according to factors such as particle number, absolute protein mass, and animal weight. Four studies used the number of EVs particles as a unit of treatment. The remaining 13 studies were divided into < 100 μg, 100 μg, and > 100 μg groups according to the injection volume for subgroup analysis. Injection doses of 100 μg (OR = 0.13, 95% CI: 0.06–0.30, P < 0.001) were found to better improve the survival rate of septic animal models (Additional file 1: Fig. S6). Additionally, EVs that were xenogenic (OR = 0.18, 95% CI: 0.09–0.36, P < 0.001) were found to be less effective than those that were allogeneic (OR = 0.15, 95% CI: 0.08–0.28, P < 0.001) (Additional file 1: Fig. S7). Compared to EVs treatment with fewer than five days of observation (OR = 0.13, 95% CI: 0.04–0.44, P < 0.001), EVs treatment with observation exceeding five days (OR = 0.12, 95% CI: 0.07–0.23, P < 0.001) could also improve animal survival. The survival rate of septic animals treated with EVs was higher when observation time exceeded 5 days (OR = 0.12, 95% CI: 0.07–0.23, P < 0.001) (Additional file 1: Fig. S8). Subgroup analysis also showed that antibiotic rehydration (OR = 0.13, 95% CI: 0.04–0.44, P < 0.001) after the establishment of sepsis models improved animal survival (Additional file 1: Fig. S9).

### Publication bias

Funnel plots were used to assess potential bias among the studies included in the meta-analysis. Additional file 1: Fig. S10 shows that no publication bias was found.

## Discussion

In this manuscript, existing preclinical EVs research was summarized and analyzed by applying meta‐research methods to summarize the efficacy of EVs in the treatment of sepsis in animals. Comprehensive analysis confirmed that MSC-EVs treatment may improve the survival rate of sepsis animal models, which provides important clues for human clinical trials. However, more studies are needed to confirm the optimal therapeutic effect of EVs on sepsis.

The SYRCLE tool was used to assess the methodological quality of the 17 studies included in this meta-analysis. In the evaluation process, a lack of clear method description was noted in the methods of many studies, as well as potential bias due to insufficient randomization and lack of blinding. Issues such as these suggest that detailed and specific method description is crucial when conducting experiments. In addition, this review suggests an urgent need for more standardized EVs characterization in preclinical studies of EVs. Providing more comprehensive experimental details will be more conducive to subsequent related research.

In recent years, EVs have gradually become a safe, feasible alternative for cell therapy due to advantages such as high stability, good permeability, and low immunogenicity and cytotoxicity [41]. EVs are thought to be released from any cell and are involved in the intercellular transmission of information in physiological and pathological processes by transferring their components (such as proteins, miRNAs, mRNAs, and mitochondria) [42, 43]. Some recent studies have also shown that EVs can inhibit the inflammatory response in the pathological process of sepsis by delivering miRNA, lncRNA or cytokines. For example, Guang et al. [44] found that the functional delivery of endothelial progenitor cell-derived extracellular vesicles to lncRNA TUG1 could improve sepsis induced by bacterial outer membrane vesicles by endowing anti-inflammatory macrophages with polarization by impeding miR-9-5p targeting SIRT1 inhibition. Another study found that mesenchymal stromal cell-derived EVs inhibits cytokine release and inflammatory responses during sepsis [17]. However, Yuhan et al. showed that erythrocyte-derived EVs aggravate inflammation, which may be a potential risk factor for transfusion-related immune regulation [34]. So far, there is no consensus on the therapeutic effect of EVs.

This article is the first meta-analysis to summarize the efficacy of EVs in treating sepsis. Animal species, models and interventions were classified for subgroup analysis. Our results show that rats treated with EVs without restriction of animal sex are more effective, while Xue-Yi et al. 's meta-analysis showed that MSCs from male mice had a better effect on sepsis [45]. This may be related to differences in the treatment of MSCs and EVs. More experiments are needed to determine why.

Subgroup analysis also showed that EVs derived from various stem cells were superior to EVs derived from other cells, which may be related to their lower immunogenicity and higher immunomedulatory capacity [46]. From the analysis, we can see that different studies used different EVs doses and dose units. Even if the meta-analysis results suggest that 100 μg may have a better therapeutic effect, there is an urgent need to compare the efficacy of different doses of EVs in the treatment of sepsis. EVs of the same species are more effective than xenografts, which is related to the highly acute rejection caused by natural antibodies and complements [47]. However, the results of subgroup analysis are not yet illustrative, as some subgroups have insufficient references. In addition, although the included studies all referred to injecting EVs into animals after re-suspending EVs with a solution of PBS, none of the studies evaluated the delivery efficiency of EVs, which is an important part of assessing the effectiveness of EVs in the treatment of sepsis. Therefore, future research on EVs treatment needs to standardize the description of EVs in more details for further summary.

## Limitations

Several potential limitations of this meta-analysis should be considered. Firstly, the current animal models of EVs in the treatment of sepsis mainly use small animals such as rats and mice, which may overestimate the effect of EVs in the treatment of sepsis. Secondly, studies with positive results are more likely to be published leading to publication bias. Third, the sample size of the included studies is all small, and larger sample size studies are needed to prove the therapeutic effect of EVs on sepsis. Finally, the use of Engauge software to extract data from the survival curve of the article may cause deviations from the original data.

## Conclusion

This meta-analysis showed that MSC-EVs treatment may be associated with lower mortality in animal models of sepsis, setting important future directions for EVs treatment of sepsis. Subsequent preclinical studies will need to address the standardization of dose, source, and timing of EVs to provide comparable data. In addition, the effectiveness of EVs in treating sepsis must be studied in large animal studies to provide important clues for human clinical trials.

## Supplementary Information

**Additional file 1.** The detailed search strategy. **Figure S1.** Forest plot summarizing the association between animal species (rat vs. mouse) and mortality in an EVs-treated sepsis model. **Figure S2.** Forest plot summarizing the association between animal gender and mortality in an EVs-treated sepsis model. **Figure S3.** Forest plot summarizing the association between sepsis models (CLP and non-CLP) and mortality after EVs treatment. **Figure S4.** The forest plot summarizes the relationship between EVs sources and mortality in the sepsis model. **Figure S5.** The forest plot summarizes the relationship between the route of EVs administration (intravenous and intratracheal) and mortality in a sepsis model. **Figure S6.** Forest plot summarizes the relationship between EVs dose and mortality in a sepsis model. **Figure S7.** Forest plot summarizing the association between EVs species (allogeneic and xenogenic) and mortality in sepsis models. **Figure S8.** Forest plot summarizing the relationship between observation days and mortality in a sepsis model treated with EVs. **Figure S9.** Forest plot to summarizing the relationship between fluid rehydration and mortality in a sepsis model treated with EVs. **Figure S10.** Forest maps test for publication bias.

## Data Availability

All data generated or analyzed during this study are included in this published article.

## Abbreviations

EVs: Extracellular vesicles
MSCs: Mesenchymal stem cells
SYRCLE: Systematic Review Centre for Laboratory Animal Experimentation
PRISMA: Preferred Reporting Items for Systematic Reviews and Meta-Analyses
