# Supplementary material for: Protective properties of extracellular vesicles in sepsis models: a systematic review and meta-analysis of preclinical studies
Source: J Transl Med. 2023 Apr 17;21:262. doi: 10.1186/s12967-023-04121-7 (PMC10108460; doi:10.1186/s12967-023-04121-7)
Supplement: Supplementary file 1 — Additional file 1. The detailed search strategy. Figure S1. Forest plot summarizing the association between animal species (rat vs. mouse) and mortality in an EVs-treated sepsis model. Figure S2. Forest plot summarizing the association between animal gender and mortality in an EVs-treated sepsis model. Figure S3. Forest plot summarizing the association between sepsis models (CLP and non-CLP) and mortality after EVs treatment. Figure S4. The forest plot summarizes the relationship between EVs sources and mortality in the sepsis model. Figure S5. The forest plot summarizes the relationship between the route of EVs administration (intravenous and intratracheal) and mortality in a sepsis model. Figure S6. Forest plot summarizes the relationship between EVs dose and mortality in a sepsis model. Figure S7. Forest plot summarizing the association between EVs species (allogeneic and xenogenic) and mortality in sepsis models. Figure S8. Forest plot summarizing the relationship between observation days and mortality in a sepsis model treated with EVs. Figure S9. Forest plot to summarizing the relationship between fluid rehydration and mortality in a sepsis model treated with EVs. Figure S10. Forest maps test for publication bias. [file 12967_2023_4121_MOESM1_ESM.doc]

**Additional file 1:**

**The detailed search strategy.**

The Detailed Search Strategy

Pubmed and Web of Science

("Sepsis"[MeSH Terms] OR "sepsis*"[All Fields]) AND ("Extracellular Vesicles"[MeSH Terms] OR ("Extracellular"[All Fields] AND "vesicles*"[All Fields]) OR ("EV"[All Fields] OR "exosome*"[All Fields] OR ("cell derived microparticles"[MeSH Terms] OR ("cell derived"[All Fields] AND "microparticles"[All Fields]) OR "cell derived microparticles"[All Fields] OR "microvesicle"[All Fields] OR "microvesicles"[All Fields])))

Cochrane Library

#1 (sepsis): ti,ab,kw

#2 MeSH descriptor: [sepsis] explode all trees

#3 #1or#2

#4 MeSH descriptor: [Extracellular Vesicles] explode all trees

#5 (Extracellular Vesicles): ti,ab,kw

#6 (EV): ti,ab,kw

#7 (exosome): ti,ab,kw

#8 (microvesicle): ti,ab,kw

#9 #4or#5or#6#7or#8

#10 #3 and #9


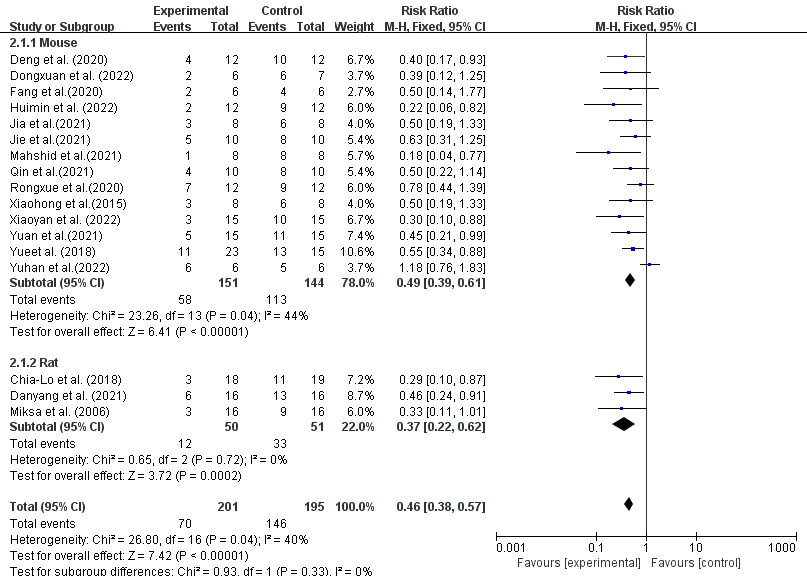


**Fig. S1**: Forest plot summarizing the association between animal species (rat vs. mouse) and mortality in an EVs-treated sepsis model.


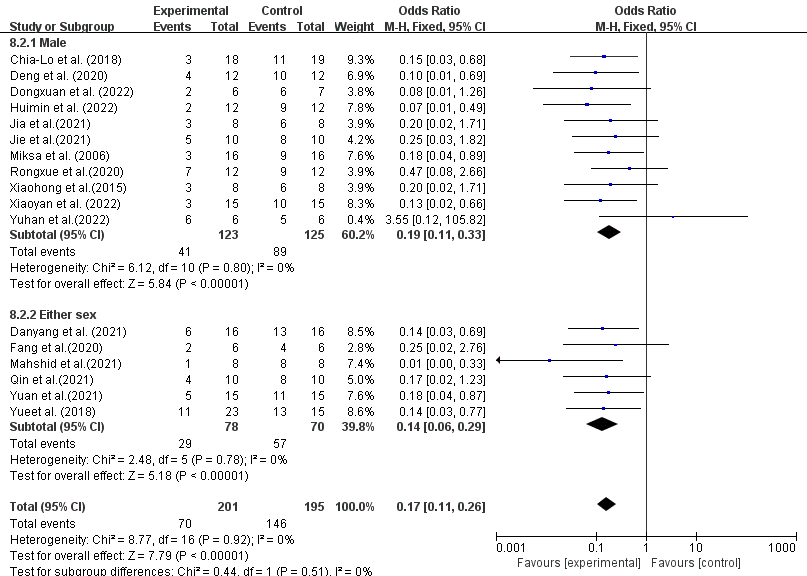


**Figure S2**：Forest plot summarizing the association between animal gender and mortality in an EVs-treated sepsis model.


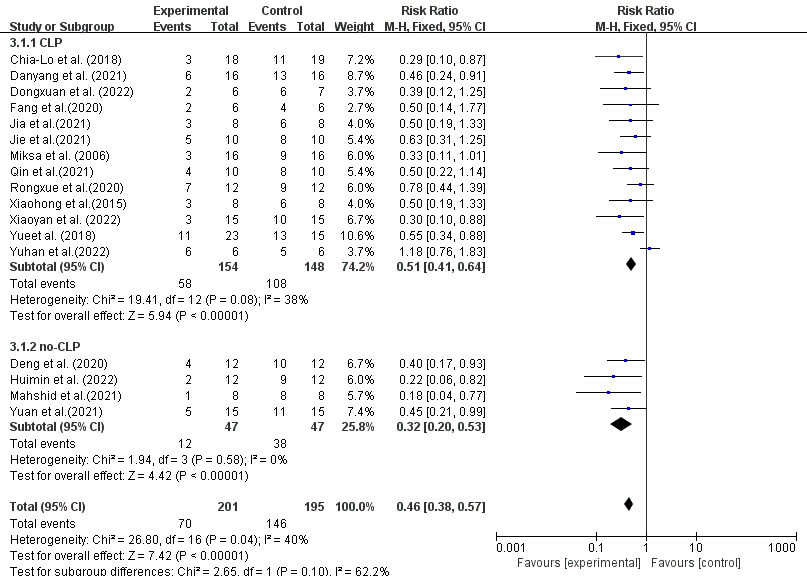


**Figure S3**：Forest plot summarizing the association between sepsis models (CLP and non-CLP) and mortality after EVs treatment.


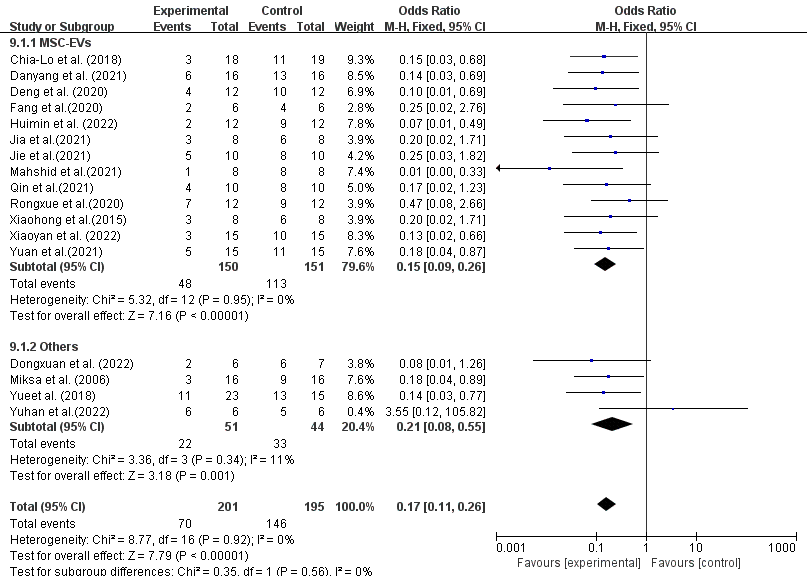


**Figure S4**：The forest plot summarizes the relationship between EVs sources and mortality in the sepsis model.


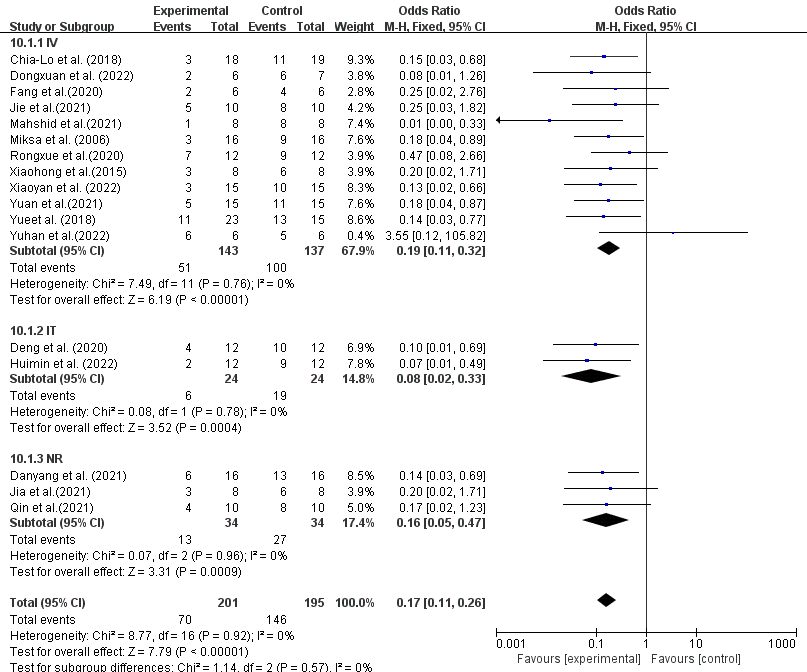


**Figure S5**:The forest plot summarizes the relationship between the route of EVs administration (intravenous and intratracheal) and mortality in a sepsis model.


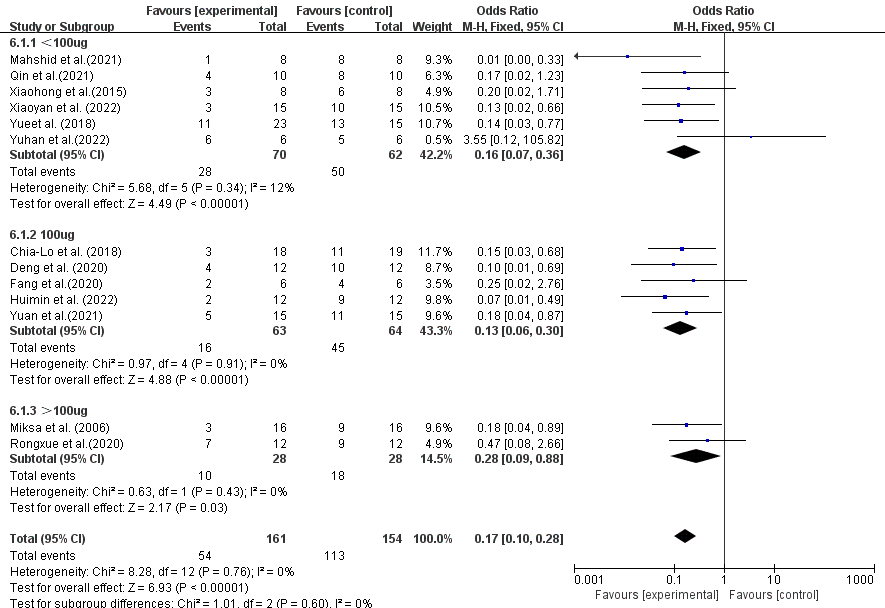


**Figure S6**:Forest plot summarizes the relationship between EVs dose and mortality in a sepsis model.


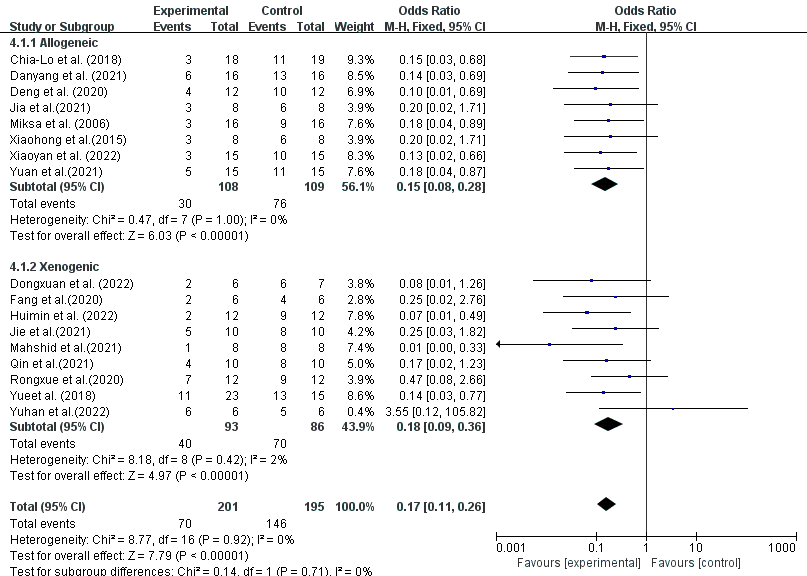


**Figure S7**: Forest plot summarizing the association between EVs species (allogeneic and xenogenic) and mortality in sepsis models.


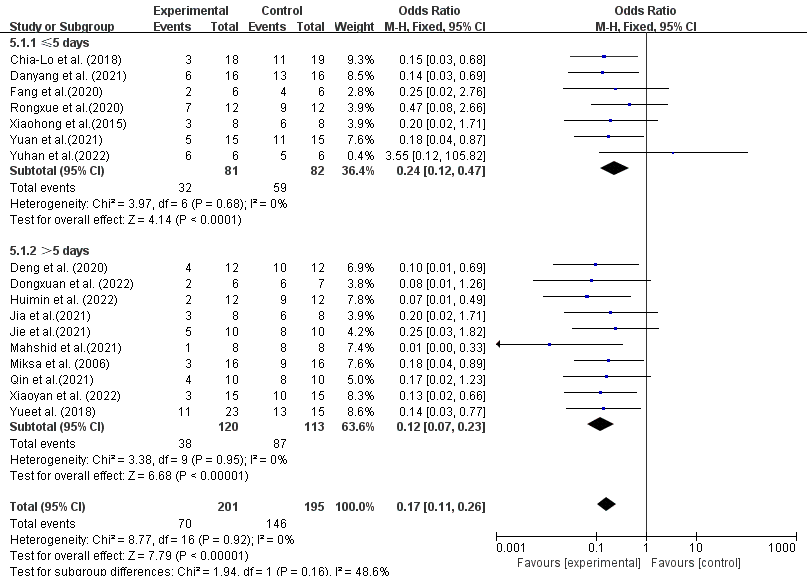


**Figure S8**:Forest plot summarizing the relationship between observation days and mortality in a sepsis model treated with EVs.


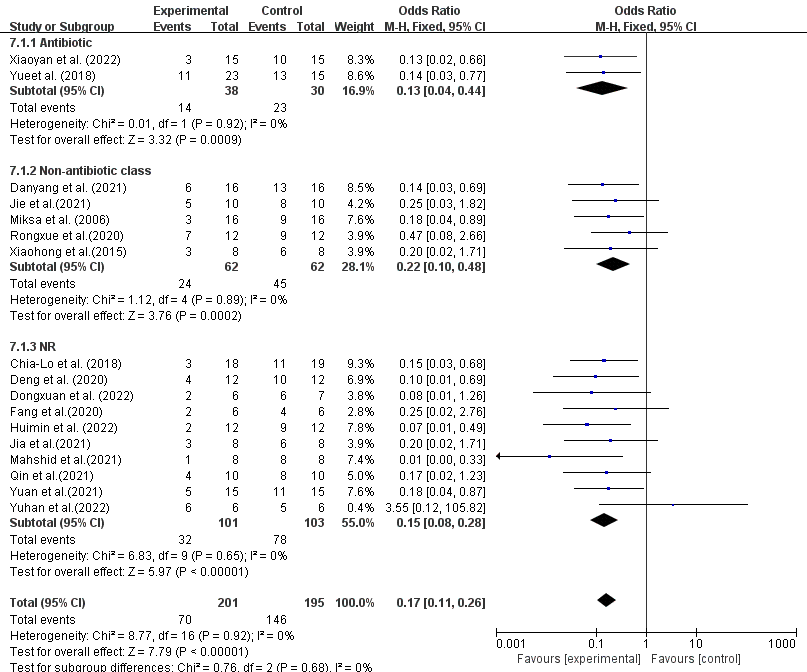


**Figure S9**:Forest plot to summarizing the relationship between fluid rehydration and mortality in a sepsis model treated with EVs.


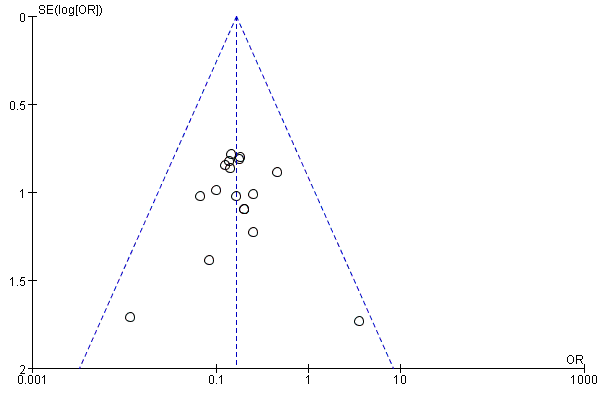


**Figure S10:**Forest maps test for publication bias.
